# Supplementary material for: Household Flood Severity and Migration Extent in Central Java: Analysis of the Indonesian Family Life Survey
Source: Int J Environ Res Public Health. 2023 May 2;20(9):5706. doi: 10.3390/ijerph20095706 (PMC10178179; doi:10.3390/ijerph20095706)
Supplement: Supplementary file 1 [file ijerph-20-05706-s001.zip › ijerph-2168195-supplementary.pdf]

Table S1. Survey questions and derived variables.

Survey book & module	Question	Response options	Measure	Derived indicator/ variable
Book 2 Household Economy / section ND Natural Disasters	ND01. In the last 5 years, was there any natural or other disaster (including civil strife) in the area where you live? If yes, what type of disasters?	○ Flood<br>○ Landslide/mudslide<br>○ Mudflow<br>○ Volcanic eruption<br>○ Earthquake<br>○ Tsunami<br>○ Windstorm<br>○ Forest fire<br>○ Fire<br>○ Civil strife<br>○ The death of Head of Household/main breadwinner<br>○ Other Household Member Deaths<br>○ Serious illness suffered by KRT/main breadwinner who require hospital care of treatment<br>○ Suffered Serious illnesses that require treatment or hospital care<br>○ Job loss or business failure experienced by Household Members<br>○ Failed Harvests<br>○ Reduction in income due to crop failure or a decrease in production rate<br>○ Drought<br>○ NONE	Types of disasters and multiple disasters	Flood severity defined as<br>○ Did not experience a flood<br>○ Experienced a flood but did not have severe impacts<br>○ Experienced a flood and had severe impacts
	ND02. Did any of the disaster was severe enough to cause death or major injuries of a household member, cause direct financial loss to the household, or cause household member to relocate?	○ Yes<br>○ No	Severity	
	ND06. Beside that disaster, what was the other disaster occurred at that time?	(Same as ND01)	Multiple disasters at the same time	
	ND11. Was any member of the household died/killed or lost because of […]	○ No one<br>○ ___ household members	Impacts	
	ND12. Did any member of the household suffer serious injury or illness because of […]	○ No one<br>○ ___ household members		

	ND14. Was the house where you were living at the time the […] disaster damaged or destroyed?	○ Not damaged<br>○ Lightly damaged<br>○ Heavily damaged<br>○ Destroyed		
	ND16. Did you receive any assistance from government and non-government organizations? (exclude family and friends) If yes, from whom?	○ Central government<br>○ Regional government<br>○ Religious groups<br>○ Political organizations<br>○ Other domestic NGOs<br>○ Private donors<br>○ Firms/corporations<br>○ Foreign government/NGO/donors<br>○ Not received assistance		
	ND18. After the […] disaster, did any member of your household spend any time living without housing, or in temporary housing?	○ Yes<br>○ No		
	ND21 Have you returned or do you expect to return?	○ Yes<br>○ No, but plan to return<br>○ No, do not plan to return		
NA[1]	NA	NA	Migration	Extent household moved[2] defined as<br>○ Did not move<br>○ Moved within the same village<br>○ Moved within the same district<br>○ Moved within the same regency<br>○ Moved within the same province<br>○ Moved to other IFLS province<br>○ Moved to other province

[1] Not part of the household survey, information was gathered through the survey's tracking of panel households. [2] This variable was generated by IFLS from the household tracking forms and provided to users in the 'htrack' data file.
